# Supplementary figures and images for: Comparative therapeutic strategies for preventing aortic rupture in a mouse model of vascular Ehlers-Danlos syndrome
Source: PLoS Genet. 2022 Mar 4;18(3):e1010059. doi: 10.1371/journal.pgen.1010059 (PMC8926273; doi:10.1371/journal.pgen.1010059)

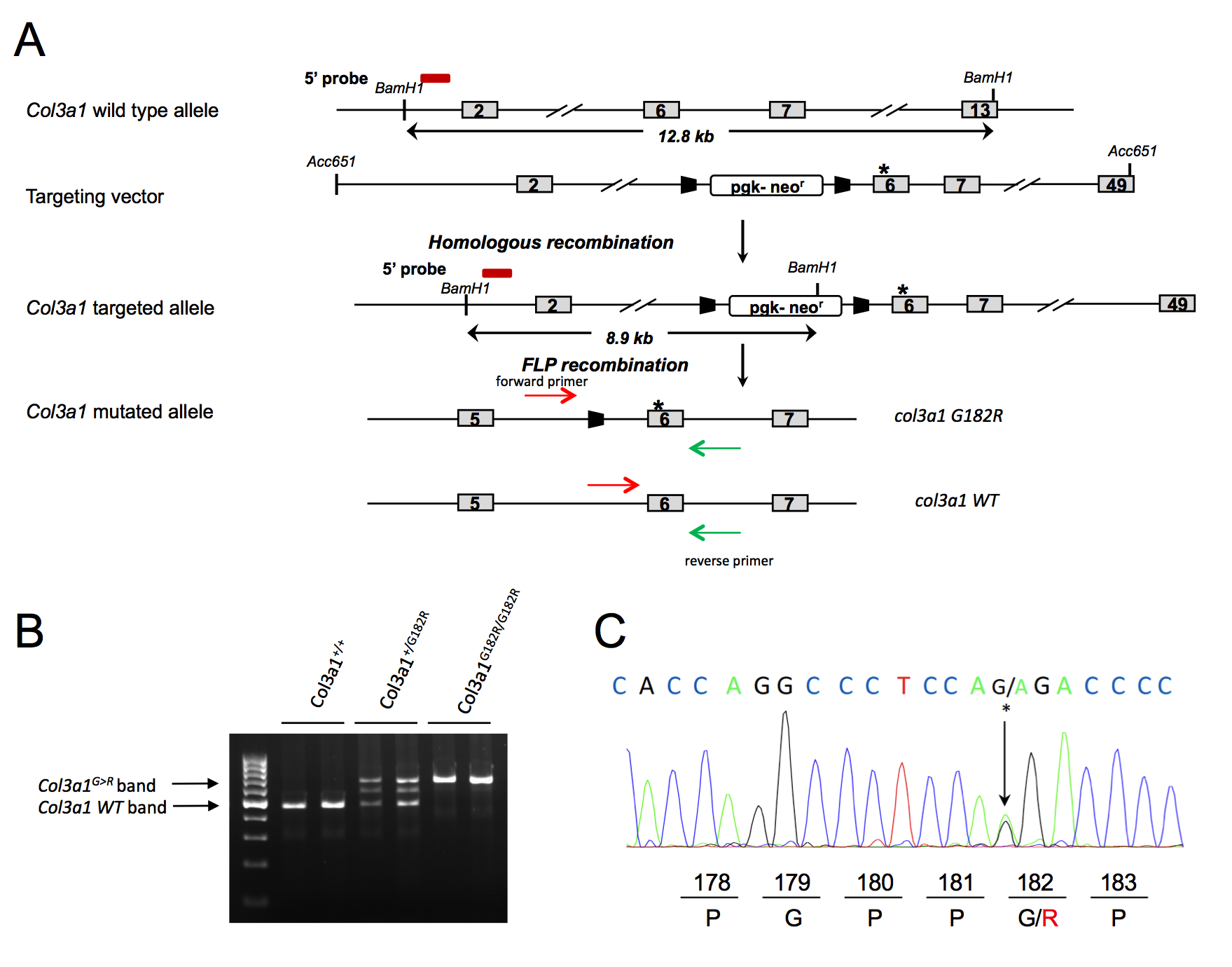

Supplement: S1 Fig — A. Gene targeting strategy used for generating the Col3a1+/G182R mutated allele. The WT and targeted alleles are shown before and after FLP recombination (with and without the pgk-neorcassette respectively). Boxes represent exons (the number of exon is indicated) and those with pgk-neor represent the neomycin selection cassette. Black boxes correspond to FRT recombination sites, the asterisk represents the mutated site in exon 6. For Southern blot analysis, the 5’ probe used is shown as a red rectangle upper the Col3a1 WT and targeted allele and relevant restrictions sites are shown (BamH1). For genotype analysis, the col3a1 reverse (green arrows) and forward (red arrows) primers are shown on both WT and mutated allele. B. The G to A substitution is checked in ES cell clones by Sanger sequencing. Compared with WT ES cell clones, heterozygous ES cell clones reveal the substitution c.547G>A leading to an amino-acid change p.G182R. The asterisk represents the mutated nucleotide on exon 6. C. Genotype analysis of WT and Col3a1 mutated mice. Both WT and mutated alleles were detected by PCR using Col3a1 primers. (TIFF) [file pgen.1010059.s001.tiff]

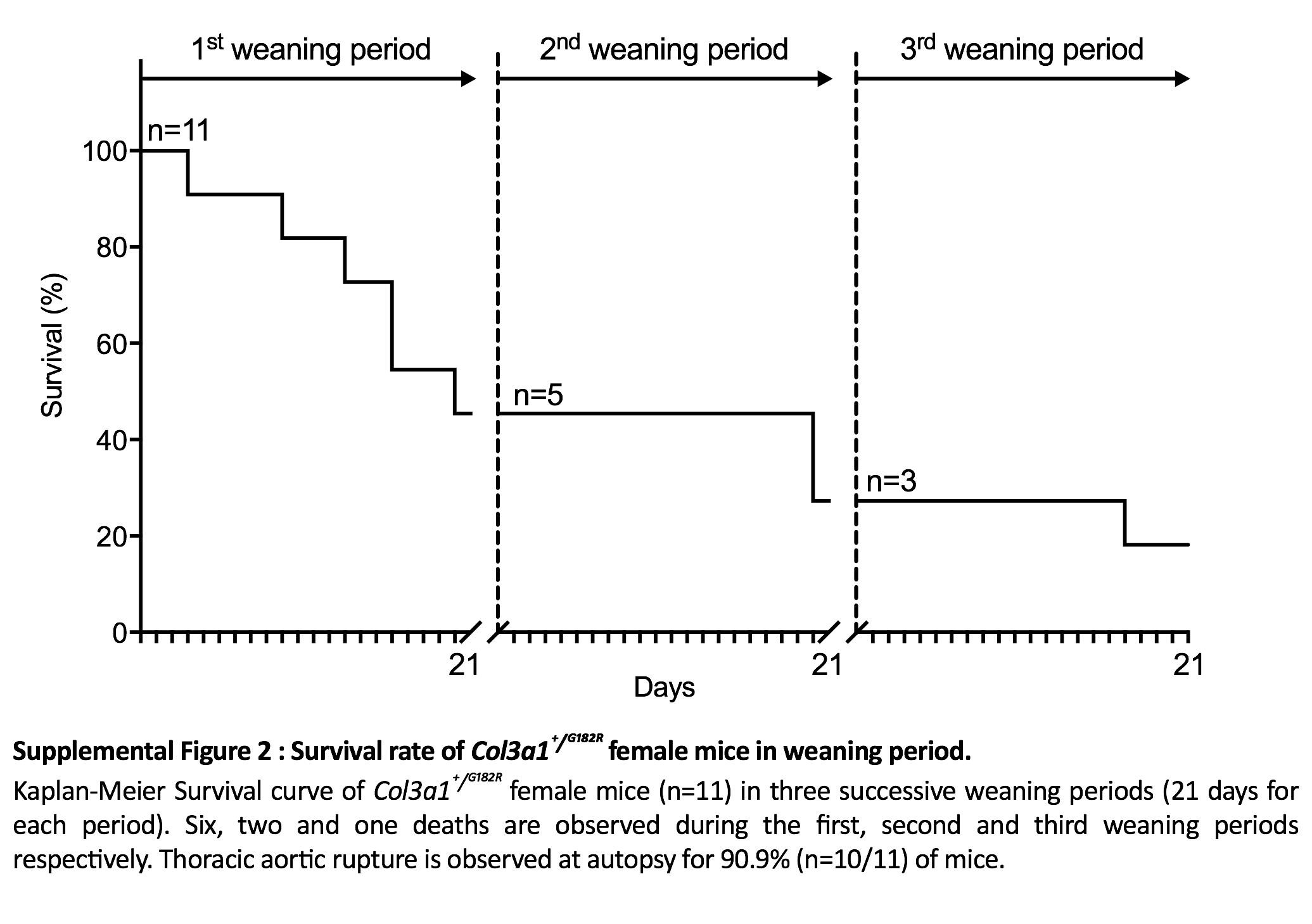

Supplement: S2 Fig — Kaplan-Meier Survival curve of Col3a1+/G182R female mice (n = 11) in three successive weaning periods (21 days for each period). Six, two and one deaths are observed during the first, second and third weaning periods respectively. Thoracic aortic rupture is observed at autopsy for 90.9% (n = 10/11) of mice. (TIFF) [file pgen.1010059.s002.tiff]

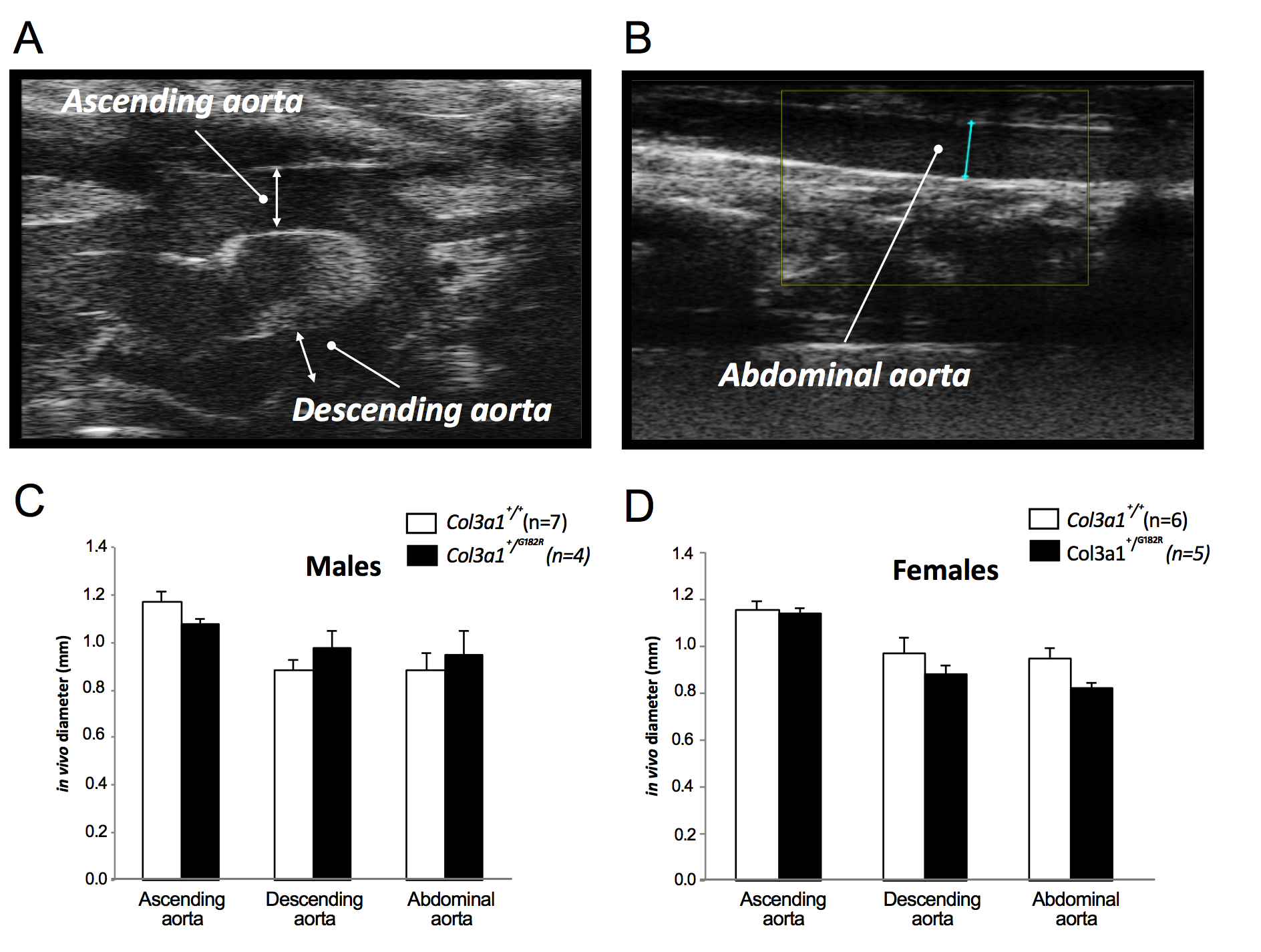

Supplement: S3 Fig — A. Ascending and descending thoracic aorta in Col3a1+/G182R. White arrows indicate the ascending and descending thoracic aortic diameter. B. Abdominal aorta in Col3a1+/G182R. Blue arrow indicates the abdominal aortic diameter. C-D. Measurement of the three diameters in males and females respectively. Error bars show mean ± SEM. No significant differences were found using Student t-test when the three diameters were compared between Col3a1+/G182R and Col3a1+/+ mice in both sexes (p>0.05). (TIFF) [file pgen.1010059.s003.tiff]

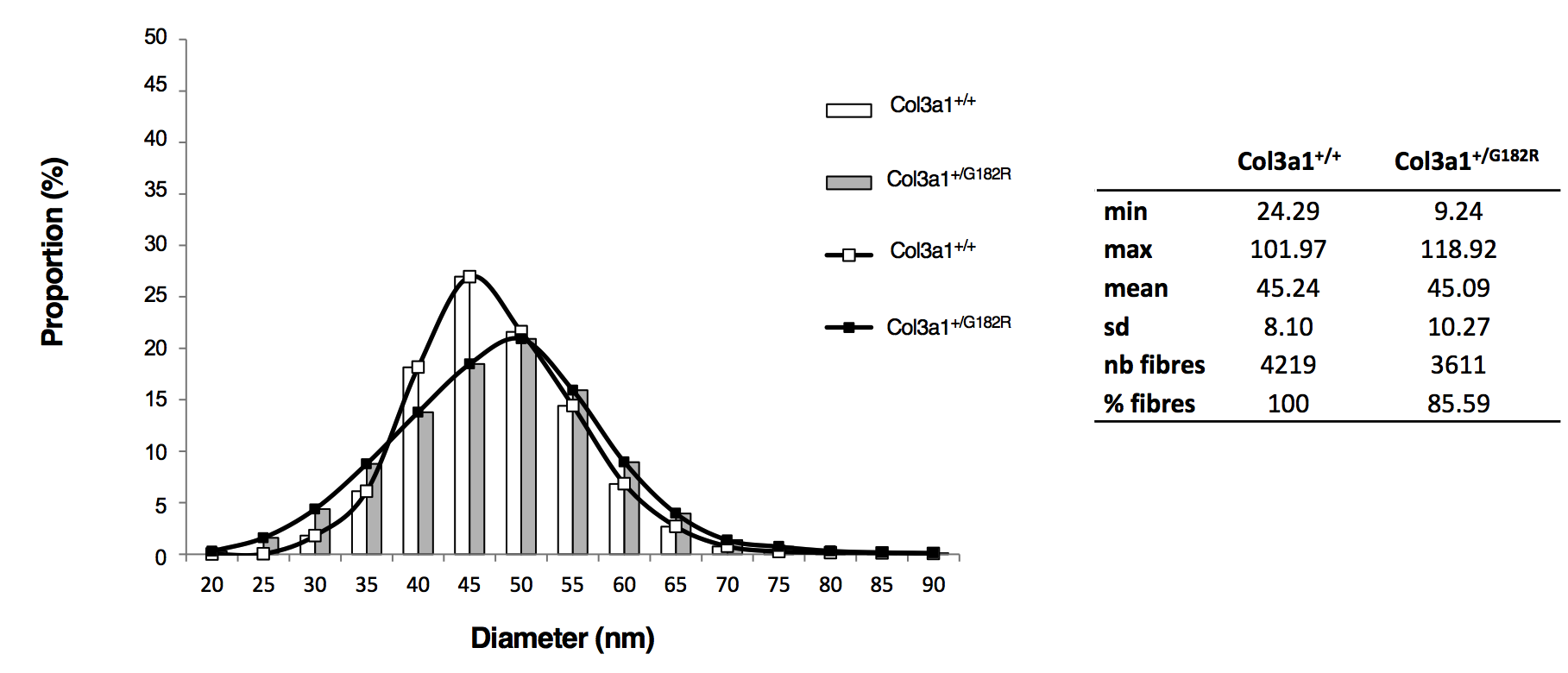

Supplement: S4 Fig — The proportion (%) of collagen fibrils with a given diameter (nm) shows heterogeneity in the distribution for Col3a1+/G182R mice compared to Col3a1+/+ controls in the aorta. In the table, the diameters of the collagen fibrils show a wider range in Col3a1+/G182R mice than in Col3a1+/+ mice. The % fibers represent the number of collagen fibrils per unit area considering the number of collagen fibrils in Col3a1+/+ mice as the reference. The % fibers reveal a lower density of collagen fibrils in Col3a1+/G182R mice than in Col3a1+/+ mice. (TIFF) [file pgen.1010059.s004.tiff]

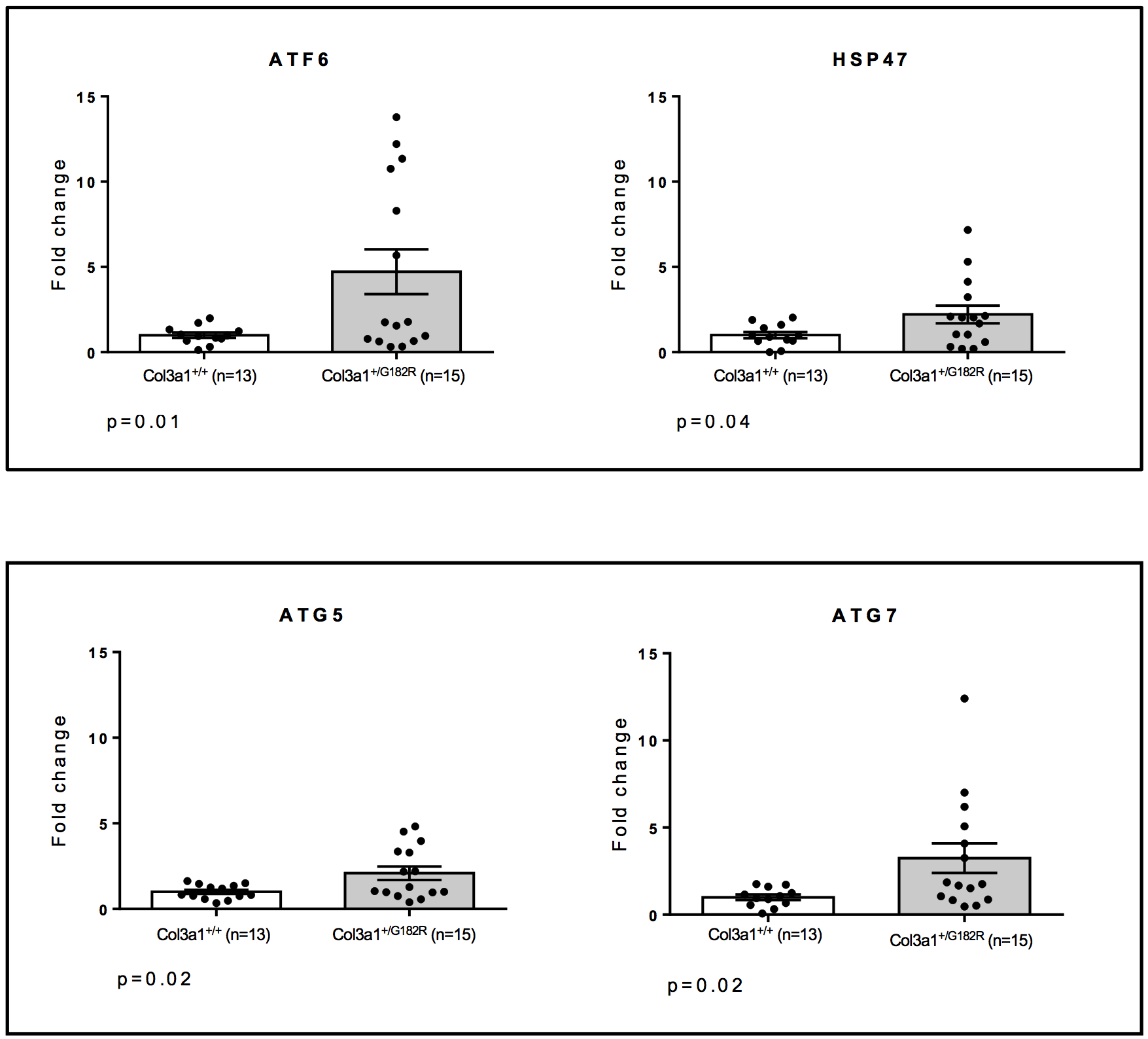

Supplement: S5 Fig — The expression of HSP47, ATF6, ATG5 and ATG7 is significantly different in the thoracic aorta of Col3a1+/G182R mice compared to Col3a1+/+ controls (Student t-test, p<0.05). (TIFF) [file pgen.1010059.s005.tiff]

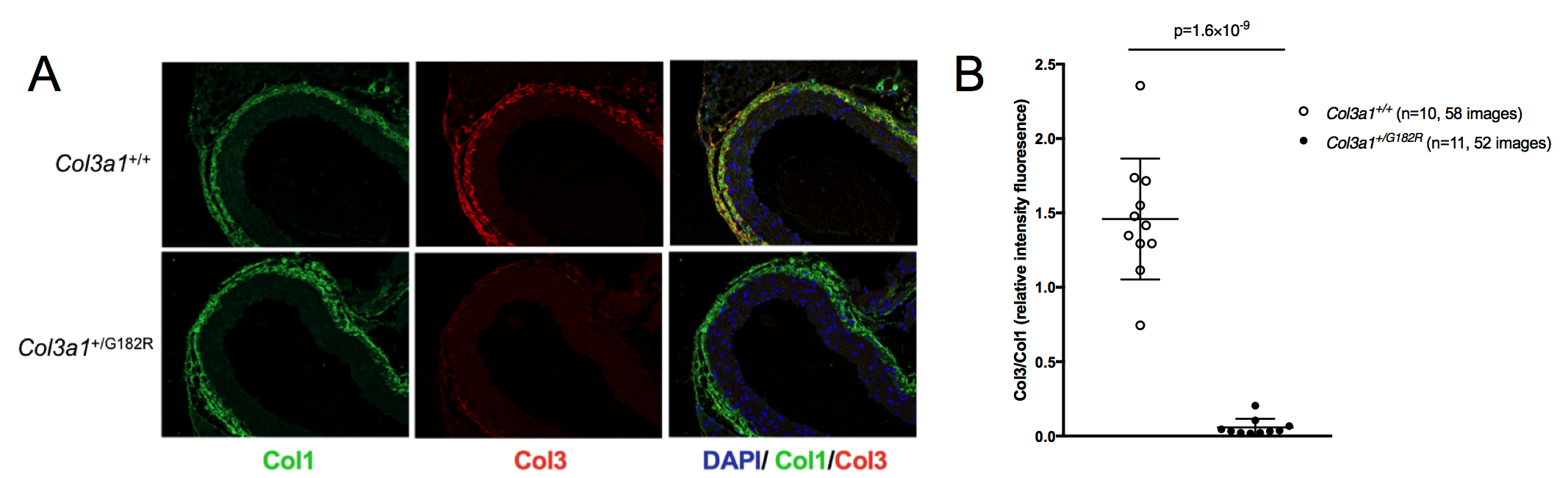

Supplement: S6 Fig — A. Immunofluorescent staining of descending TA sections of Col3a1+/+ (n = 10) and Col3a1+/G182R (n = 10) mice to show the distribution of collagens I and III. The sections are stained with collagen I (green) and collagen III (red) polyclonal antibodies and the cell nuclei were stained with DAPI (blue). The anti-collagen III antibody failed to recognize mature collagen III in Col3a1+/G182R mice. B. Relative quantification of the mature collagen III using the Collagen III/Collagen I immunofluorescence intensity ratio: the quantity of collagen III was collapsed probably due to conformational changes which lead to the absence of detection of collagen III, in the TA of Col3a1+/G182R mice compared to Col3a1+/+ controls (Wilcoxon test, p<10−4). (TIFF) [file pgen.1010059.s006.tiff]

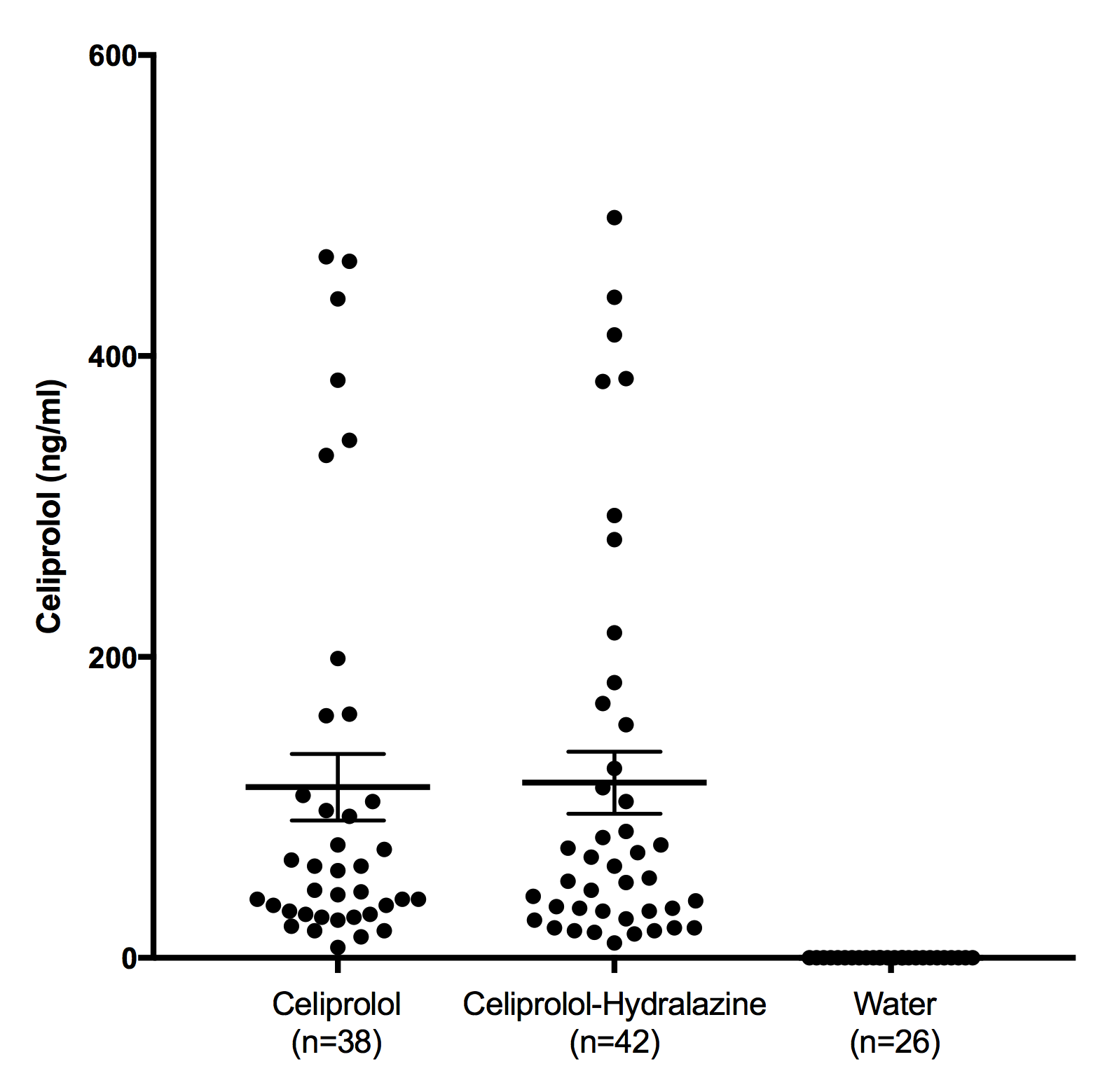

Supplement: S7 Fig — Plasma concentration of celiprolol in Col3a1+/G182R (n = 80) mice receiving celiprolol or the association celiprolol-hydralazine and in Col3a1+/G182R (n = 26) mice receiving water, all of them being included in the celiprolol and celiprolol-hydralazine protocols. The celiprolol concentration was not significantly difference between the two treated groups. Data are expressed as the mean ± SEM. (TIFF) [file pgen.1010059.s007.tiff]

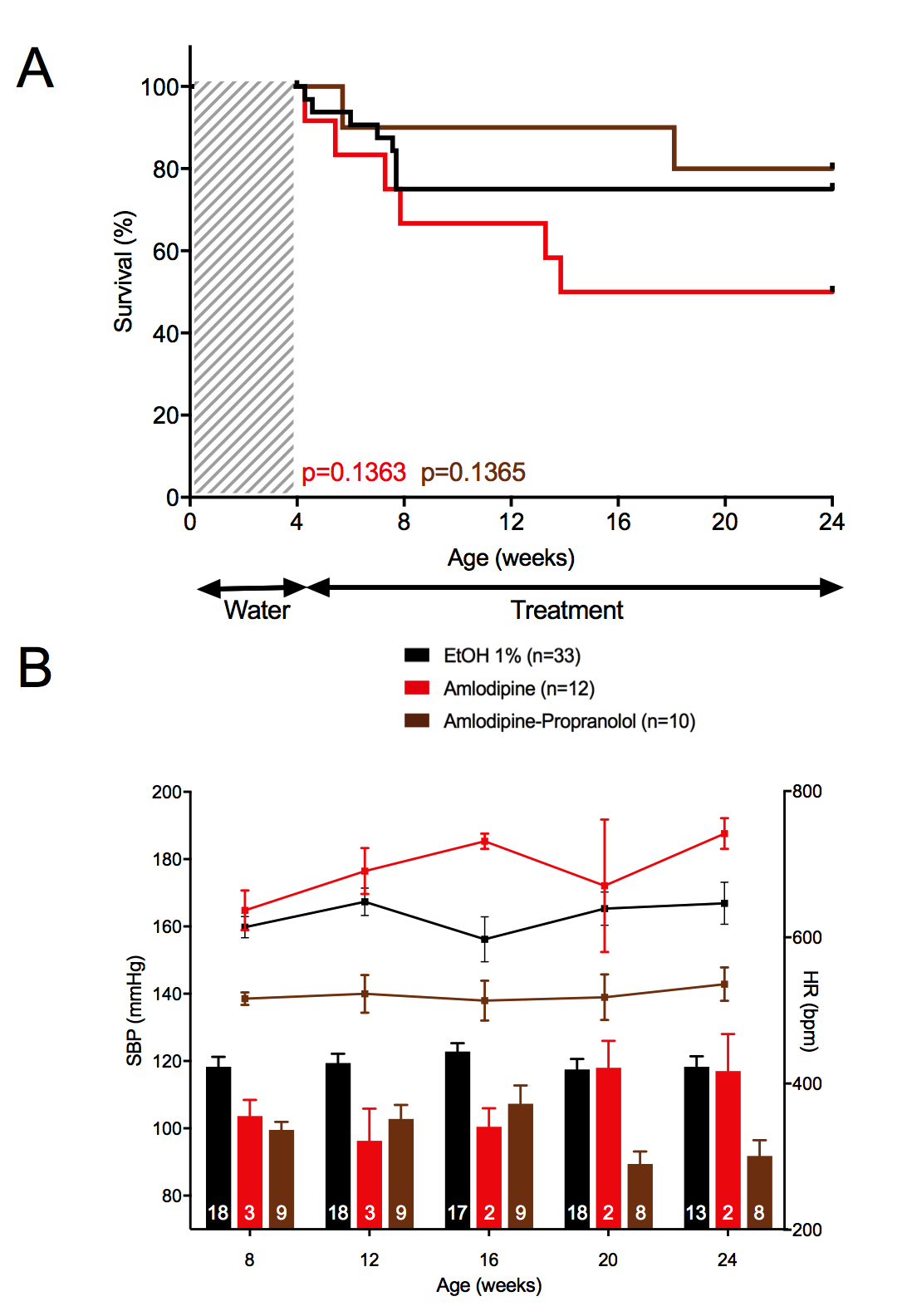

Supplement: S8 Fig — A. Survival comparison between Amlodipine and ethanol 1%, and between the association amlodipine-propranolol and amlodipine (monotherapy). Kaplan-Meier Survival curve (red curve) for comparing Col3a1+/G182R treated with amlodipine (n = 12) to Col3a1+/G182R treated with ethanol 1% (n = 33). Amlodipine worsens the mortality despite no significant difference is observed using Log-Rank (Mantel-Cox) analysis (p = 0.1363). Kaplan-Meier Survival curve (brown curve) for comparing Col3a1+/G182R treated with amlodipine-propranolol (n = 10) to Col3a1+/G182R treated with amlodipine (n = 12). The association improves slightly the survival despite insignificant difference is calculated using Log-Rank (Mantel-Cox) analysis (p = 0.1365). B. SBP and HR comparison between amlodipine and ethanol 1%, and between the association amlodipine-propranolol and amlodipine (monotherapy). A significant decrease in SBP (linear mixed-effects model, p = 8.17×10^–3^) was observed that was associated with no change in HR (student t-test, p>0.05 for each time of the 24-week follow-up period) when comparing amlodipine to ethanol 1% (red bars and curve). (TIFF) [file pgen.1010059.s008.tiff]

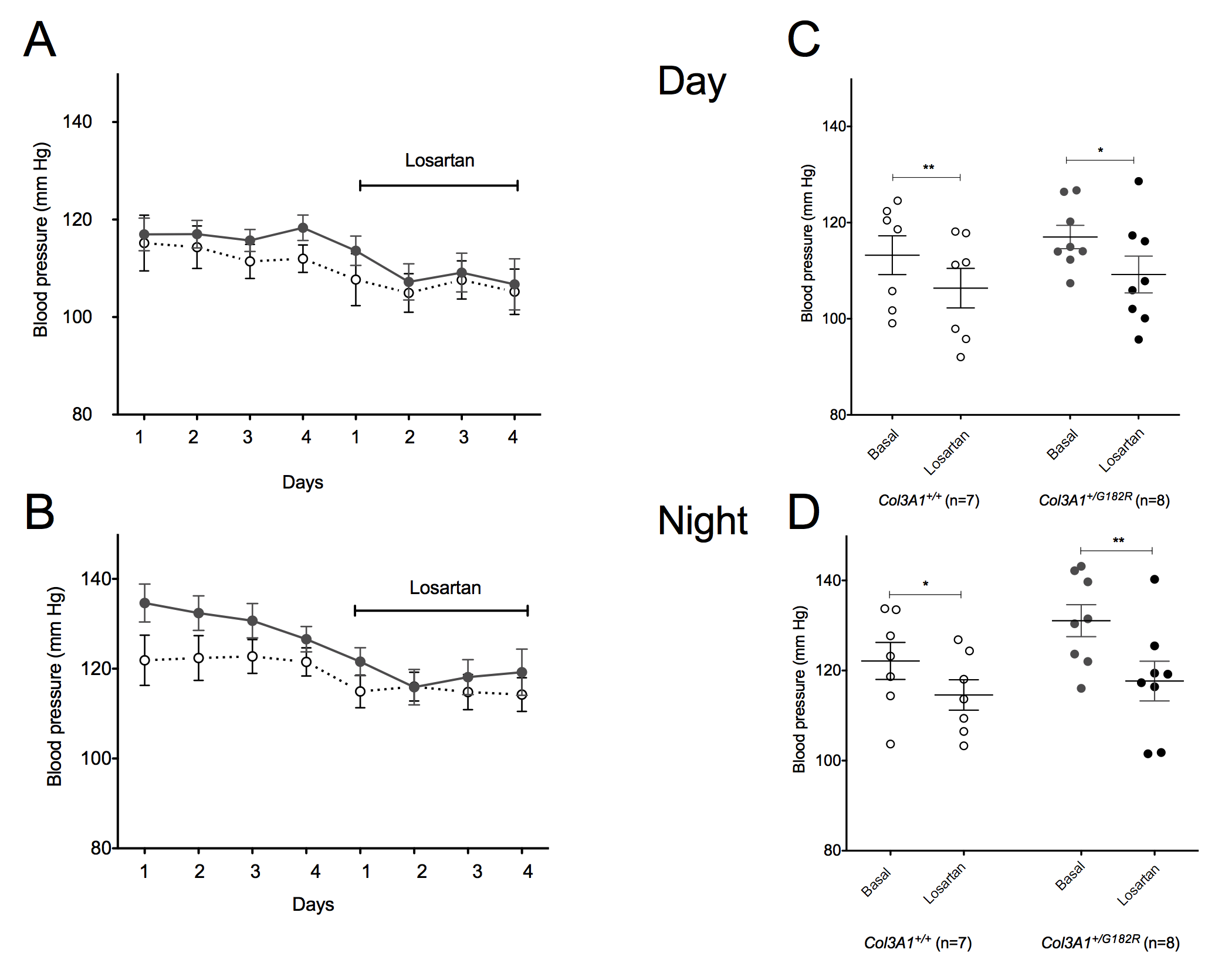

Supplement: S9 Fig — A. Day SBP before (4 days = basal) or during oral administration (4 days) of losartan (135 mg/kg/day) in Col3a1+/G182R mice (n = 8) compared to Col3a1+/+ mice (n = 7) examined with a telemetric system. B. Night SBP before (4 days = basal) or during oral administration (4 days) of losartan (135 mg/kg/day) in the same Col3a1+/G182R mice (n = 8) and Col3a1+/+ mice (n = 7). C. Comparison of the mean of the day SBP before and during administration of losartan in the same Col3a1+/G182R mice (n = 8) and Col3a1+/+ mice (n = 7). Significant decrease is observed in both groups. D. Comparison of the mean of the night SBP before and during administration of losartan in the same Col3a1+/G182R mice (n = 8) and Col3a1+/+ mice (n = 7). Significant decrease is observed in both groups. (TIFF) [file pgen.1010059.s009.tiff]

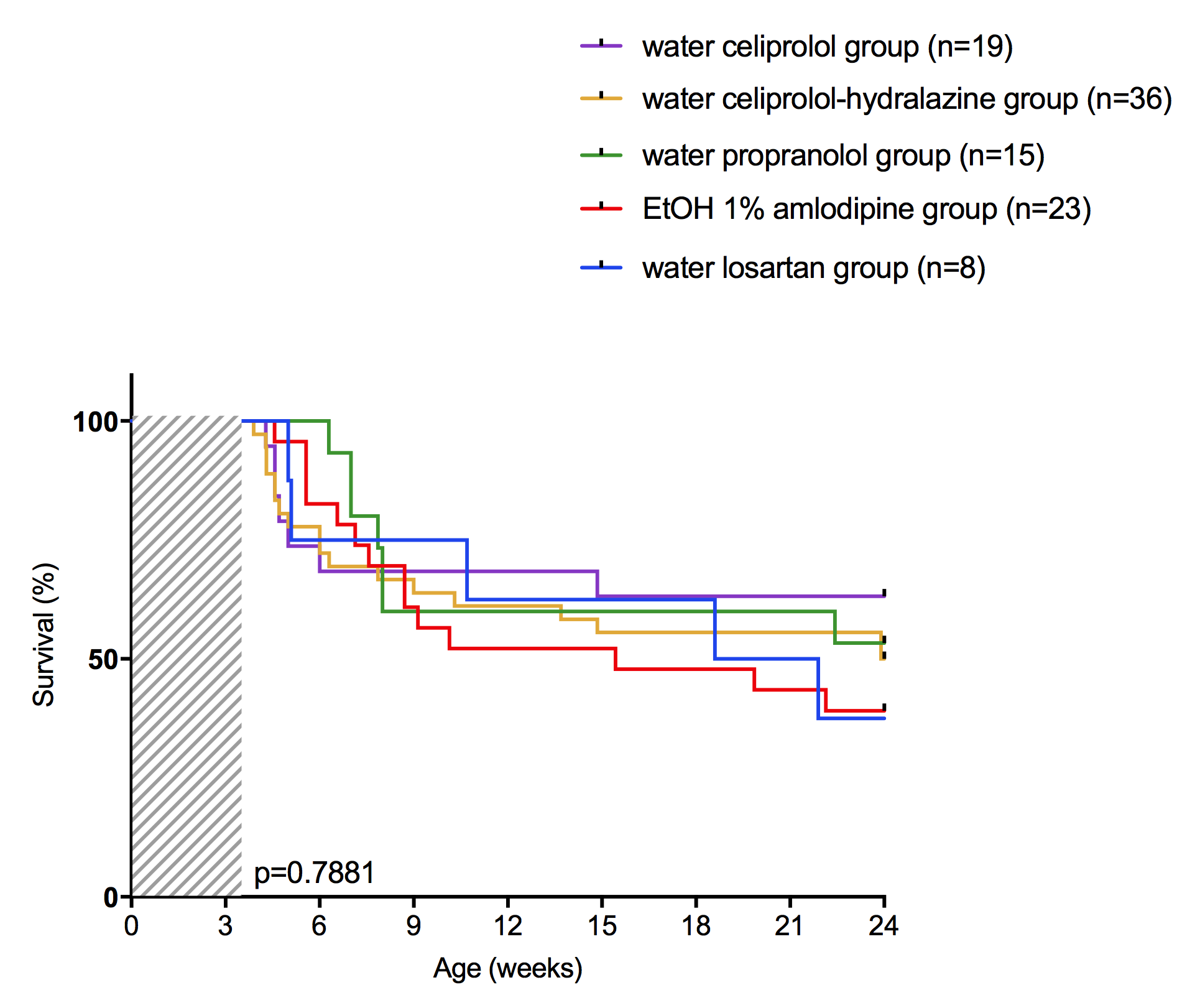

Supplement: S10 Fig — Kaplan-Meier Survival curve for comparing the 5 Col3a1+/G182R male control groups. No significant difference is observed using Log-Rank (Mantel-Cox) analysis (p = 0.7881). (TIFF) [file pgen.1010059.s010.tiff]
