# Supplementary material for: Comparative therapeutic strategies for preventing aortic rupture in a mouse model of vascular Ehlers-Danlos syndrome
Source: PLoS Genet. 2022 Mar 4;18(3):e1010059. doi: 10.1371/journal.pgen.1010059 (PMC8926273; doi:10.1371/journal.pgen.1010059)
Supplement: Attachment — Submitted filename: Response to Reviewers Col3a1KI.docx [file pgen.1010059.s020.docx]

REVIEWER COMMENTS:

*We would like to thank the reviewers for their constructive comments and interesting suggestions to improve the quality of our manuscript.*

*We have addressed all concerns of the reviewers below and all modifications are mentioned in red in the manuscript.*

**Reviewer #1:**

1. This investigation describes the phenotype of a newly created transgenic mouse line with respect to mortality, biochemical pathways, and changes in survival in response to several pharmacological manipulations. This mouse line was created to recapitulate characteristics of the vascular Ehler-Danlos syndrome observed in humans by modifying the collagen III product. In this regard, there are published reports from at least two other transgenic mouse lines that use different modifications of the collagen III gene. The authors show that their transgenic model is similar to the human condition in many important ways (as do the other studies) but does differ with respect to survival benefits in response to celiprolol, which has β1-antagonistic and β2-agonistic properties. In addition, the authors highlight some important differences in activation of certain biochemical pathways between this transgenic line and those previously published.

The experiments have been carefully thought out and the number of animals in experimental cohorts are, in general, adequate. Statistical analyses are appropriate for this set of studies – with the possible exception that changes in systolic blood pressure and heart rate may have benefitted from applying repeated measures ANOVA tests given that the same animals were tracked over time. This may have been done as part of the mixed-effects modeling; but, if that is the case, it is not explicitly stated. In addition, comparisons between groups should be corrected for the effects of multiple comparisons (Tukey’s, Bonferroni’s, or Scheffe’s tests).

*Answer: We thank the reviewer for his positive comments and advices about statistical analyses. Prior to generalized linear mixed model (GLMM), all measures were tested for repeated measures ANOVA (RMA) and a Bonferroni correction was applied, leading to the same results as GLMM. These results were given in the following tables (the p value of each comparison was indicated) and were not reported in the manuscript.*

| ***SBP*** | ***8 vs 12*** | ***8 vs 16*** | ***8 vs 20*** | ***8 vs 24*** | ***12 vs 16*** | ***12 vs 20*** | ***12 vs 24*** | ***16 vs 20*** | ***16 vs 24*** | ***20 vs 24*** |
| --- | --- | --- | --- | --- | --- | --- | --- | --- | --- | --- |
| *Propranolol* | *0.095* | *1.000* | *0.683* | *0.092* | *1.000* | *1.000* | *1.000* | *1.000* | *1.000* | *1.000* |
| *Celiprolol* | *0.776* | *0.526* | *0.715* | *0.403* | *0.517* | *0.931* | *0.232* | *0.426* | *0.605* | *0.320* |
| *Amlodipine* | *NA* | *NA* | *NA* | *NA* | *NA* | *NA* | *NA* | *NA* | *NA* | *NA* |
| *Amlodipine in females (F)* | *NA* | *NA* | *NA* | *NA* | *NA* | *NA* | *NA* | *NA* | *NA* | *NA* |
| *Amlodipine - propranolol* | *NA* | *NA* | *NA* | *NA* | *NA* | *NA* | *NA* | *NA* | *NA* | *NA* |
| *Amlodipine - propranolol in F* | *NA* | *NA* | *NA* | *NA* | *NA* | *NA* | *NA* | *NA* | *NA* | *NA* |
| *Hydralazine* | *1.000* | *1.000* | *1.000* | *1.000* | *1.000* | *1.000* | *1.000* | *1.000* | *1.000* | *1.000* |
| *Hydralazine - celiprolol* | *1.000* | *1.000* | *1.000* | *1.000* | *1.000* | *1.000* | *1.000* | *0.900* | *1.000* | *1.000* |
| *Losartan* | *0.441* | *0.935* | *0.693* | *0.827* | *0.736* | *0.450* | *0.110* | *0.589* | *0.869* | *0.805* |
| *Losartan at weaning* | *1.000* | *1.000* | *1.000* | *1.000* | *1.000* | *0.449* | *0.883* | *1.000* | *1.000* | *0.500* |

*NA: not applicable due to missing data or data that did not pass normality.*

| ***HR*** | ***8 vs 12*** | ***8 vs 16*** | ***8 vs 20*** | ***8 vs 24*** | ***12 vs 16*** | ***12 vs 20*** | ***12 vs 24*** | ***16 vs 20*** | ***16 vs 24*** | ***20 vs 24*** |
| --- | --- | --- | --- | --- | --- | --- | --- | --- | --- | --- |
| *Propranolol* | *0.724* | *1.000* | *1.000* | *1.000* | *1.000* | *1.000* | *1.000* | *1.000* | *1.000* | *1.000* |
| *Celiprolol* | *1.000* | *0.820* | *1.000* | *0.788* | *1.000* | *1.000* | *1.000* | *1.000* | *1.000* | *1.000* |
| *Amlodipine* | *1.000* | *1.000* | *0.769* | *0.069* | *1.000* | *1.000* | *1.000* | *1.000* | *0.498* | *1.000* |
| *Amlodipine in females (F)* | *NA* | *NA* | *NA* | *NA* | *NA* | *NA* | *NA* | *NA* | *NA* | *NA* |
| *Amlodipine - propranolol* | *NA* | *NA* | *NA* | *NA* | *NA* | *NA* | *NA* | *NA* | *NA* | *NA* |
| *Amlodipine - propranolol in F* | *NA* | *NA* | *NA* | *NA* | *NA* | *NA* | *NA* | *NA* | *NA* | *NA* |
| *Hydralazine* | *0.568* | *0.156* | *0.091* | *0.023* | *1.000* | *1.000* | *0.129* | *1.000* | *1.000* | *1.000* |
| *Hydralazine - celiprolol* | *1.000* | *1.000* | *0.577* | *0.266* | *1.000* | *1.000* | *0.911* | *1.000* | *1.000* | *1.000* |
| *Losartan* | *1.000* | *0.335* | *0.159* | *0.866* | *1.000* | *1.000* | *1.000* | *1.000* | *1.000* | *1.000* |
| *Losartan at weaning* | *0.336* | *1.000* | *0.090* | *1.000* | *1.000* | *1.000* | *1.000* | *1.000* | *1.000* | *0.255* |

*NA: not applicable due to missing data or data that did not pass normality.*

*However, due to the design of the study there was a non-negligible number of missing data (mortality rate of our model). RMA would only use listwise deletion, reducing power, while GLMM handles missing data better as well as differing number of repeats. In our design we had a repeated measure over time but mice were also clustered in other groupings that RMA could not incorporate while GLMM could. Moreover, Shapiro-Wilk's test showed non-normal residuals in some groups and therefore ANOVA was not an option as there is no RMA equivalent for logistic regression models. The sentence: ”Prior to generalized linear mixed model (GLMM), all measures were tested for repeated measures ANOVA (RMA) and a Bonferroni correction was applied, leading to the same results as GLMM.” was added in the materials and methods section.*

*We also modified Figure 4A by removing the line indicating the insignificance between the expression of the WT and mutated alleles in Col3a1^+/G182R^ mice because the statistical test performed compared the expression of the mutated allele to a theoretical 50% and not to the expression of the WT allele.*

2. The display analysis of the survival analysis for the lactating mothers (Figure S2) seems a little strange to be put on a continuous time scale – unless, the mothers that were alive through the pre-weaning period from the previous littler were immediately pregnant. That would suggest that these mothers may have been pregnant and lactating at the same time. If there was a gap between conceptions, it may be more fruitful to overlay the survival curves and then indicate the “number at risk” through 21 days for each of the three lactation periods.

*Answer: We made modifications to make the figure more explicit. In this experiment, as we have observed that the majority of deaths occur during lactation and not during gestation, females were included just after the first birth. Thus mothers could have been pregnant and lactating at the same time. Of the 9 observed deaths, two involved both lactating and pregnant mice (during the second lactation) and the other 7 involved lactating mice only. We chose to use a continuous time scale and not to overlay the 3 survival curves in order to show the successive lactation periods for some mice from the initial group of 11 mice. Thus, the mothers could have had a single lactation, or two successive lactations, or 3 successive lactations. We hope that the proposed new supplemental figure 2 will be more appropriate and clearer for the readers. The sentence: “Of the 9 observed deaths, two involved both lactating and pregnant mice (during the second lactation) and the other 7 involved lactating mice only.” was added in the results section.*

3. Finally, were the data for mRNA expression and immunoblotting checked for normality of distribution? If not normally distributed, please consider log transforming the data prior to running parametric tests.

*Answer: We agree with the reviewer comment and check if the data for mRNA expression and immunoblotting pass Shapiro-Wilk normality tests. All the data pass the Shapiro-Wilk normality test for the expression of total col3a1 mRNA and the quantification of western blot. Thus, we modified the corresponding paragraph in the material and methods section as follows: “When the Shapiro-Wilk normality test was passed, a student t-test for unpaired series was used to compare between Col3a1^+/G182R^ and Col3a1^+/+^ mice: the area and collagen density in the different aorta sections, the expression of total col3a1 mRNA, the quantification of western blot and qPCR results and the quantification of TGF-β isoforms. For the relative expression of collagen III by immunofluorescence that did not pass Shapiro-Wilk normality test, Wilcoxon (nonparametric) test was performed to evaluate significance between groups”.*

4. Minor comments:

Introduction: The sentence beginning on line 64 (“By far the most…”) is difficult to follow. Please consider rephrasing.

Introduction: On line 77, it may be better to state “thinner arterial walls” rather than “thinner arterial thickness”.

Introduction: On line 83, “arterial” may be deleted.

Results: Line 256: “Supplemental Figure 7” should be “Supplemental Figure 8”.

Discussion: all through: “coherence” may be better stated as “concordance” or “consistent with”.

Discussion: Line 379: “closed” should be “close”.

Methods: Line 409: Please remove the opening parenthesis before “This”.

Methods: Line 512 – “Echography” should be “ultrasound” – and also through the paragraph. If you performed any echocardiography, you have not shown the results.

Methods: Echography paragraph – please change to past tense to be consistent with the rest of the methods section.

Methods: Line 515 – “transducer” is missing after “MHz”.

*Answer: All the minor modifications were made in the manuscript and are mentioned in the corresponding paragraphs in red, except for the third requested modification concerning the BBEST study. We chose to keep the word arterial because the primary endpoints were arterial events (rupture or dissection, fatal or not) in this study that corresponded to an assessment of arterial morbidity and mortality.*

**Reviewer #2:**

Even though your research was extensive and broad in scope, I found it easy to read your manuscript and follow the work that you did. I think it should be published with haste as it will be useful for planning clinical trials in vEDS. There are just a couple improvements that would make the manuscript easier to read.

*We thank the reviewer for this positive comment.*

1. Confusing numbers Aortic samples were taken after sacrifice at 4 weeks, 6 weeks, 24 weeks, each time studying small sample numbers. At times the n (number individual aorta samples) is not written in text but only in figure. I find it helpful to have the n= number near the p value. Please provide the n number in the text itself.

*Answer: To make it clearer how many aortic samples were used for each experiment, we indicated the n (number of individual aortic samples) near the p value of the corresponding statistical test in the results section.*

2. Please also clarify or confirm that the samples taken for aortic study were from untreated animals, both wild type and heterozygous *COL3A1* mutants.

*Answer: In order to clarify that the first experiments (macroscopic analysis of aortic ruptures, collagen quantification in the aortic wall and electron microscopy) were made in basal conditions, i.e. without treatment, we added the term “untreated” in the manuscript when needed.*

3. COL3A1 protein expression: Is the type III collagen antibody used in immunoflorescence and in Western blot from the p-ter region of the molecule? I found a description of immunofluorescent antibody location only. Might this contribute to the discrepant results (increased proIII on Western and absent on immunofluorescence).

*Answer: The targets of the antibodies are indicated in the material and methods. Indeed, two different antibodies were used. The one used for immunofluorescence was produced using the whole human mature collagen III as immunogen, essentially constituted of the triple helix domain; whereas the one used for western-blot was produced using a peptide of the C-terminal propeptide of procollagen III (Asp1224-Lys1371). These two antibodies therefore do not recognize the same antigen, which may explain the discordant results obtained in western-blot and immunofluorescence, and that is why the term “procollagen III” was used in the sentence about western-blot and the term “mature collagen III” in the sentence about immunofluorescence in the results section. The sentence: “Moreover, the two antibodies do not recognize the same antigen, which may explain the discordant results obtained in western-blot and immunofluorescence.” was also added in the corresponding paragraph.*

4. Please include the p. number for the G182R in the mouse model to clarify that it is not a triple helical number.

*Answer: In order to clarify the position of the Glycine substitution and to respect the sequence variant nomenclature, we have added the p. each time the position of the substitution was mentioned in the manuscript. Indeed, the number 182 corresponds to the amino-acid position in the preprocollagen monomer and not to the amino-acid position within the triple helix domain.*
